# Inside the Noonan “universe”: Literature review on growth, GH/IGF axis and rhGH treatment: Facts and concerns

**DOI:** 10.3389/fendo.2022.951331

**Published:** 2022-08-18

**Authors:** Stefano Stagi, Vittorio Ferrari, Marta Ferrari, Manuela Priolo, Marco Tartaglia

**Affiliations:** ^1^ Department of Health Sciences, University of Florence, Anna Meyer Children’s University Hospital, Florence, Italy; ^2^ Medical Genetics Unit, Grande Ospedale Metropolitano “Bianchi-Melacrino-Morelli”, Reggio Calabria, Italy; ^3^ Genetics and Rare Diseases Research Division, Ospedale Pediatrico Bambino Gesù, IRCCS, Rome, Italy

**Keywords:** Noonan syndrome, RASopathies, growth, puberty, growth hormone, cancer, hypertrophic cardiomyopathy, genotype-phenotype correlations

## Abstract

Noonan syndrome (NS) is a disorder characterized by a typical facial gestalt, congenital heart defects, variable cognitive deficits, skeletal defects, and short stature. NS is caused by germline pathogenic variants in genes coding proteins with a role in the RAS/mitogen-activated protein kinase signaling pathway, and it is typically associated with substantial genetic and clinical complexity and variability. Short stature is a cardinal feature in NS, with evidence indicating that growth hormone (GH) deficiency, partial GH insensitivity, and altered response to insulin-like growth factor I (IGF-1) are contributing events for growth failure in these patients. Decreased IGF-I, together with low/normal responses to GH pharmacological provocation tests, indicating a variable presence of GH deficiency/resistance, in particular in subjects with pathogenic *PTPN11* variants, are frequently reported. Nonetheless, short- and long-term studies have demonstrated a consistent and significant increase in height velocity (HV) in NS children and adolescents treated with recombinant human GH (rhGH). While the overall experience with rhGH treatment in NS patients with short stature is reassuring, it is difficult to systematically compare published data due to heterogeneous protocols, potential enrolment bias, the small size of cohorts in many studies, different cohort selection criteria and varying durations of therapy. Furthermore, in most studies, the genetic information is lacking. NS is associated with a higher risk of benign and malignant proliferative disorders and hypertrophic cardiomyopathy, and rhGH treatment may further increase risk in these patients, especially as dosages vary widely. Herein we provide an updated review of aspects related to growth, altered function of the GH/IGF axis and cell response to GH/IGF stimulation, rhGH treatment and its possible adverse events. Given the clinical variability and genetic heterogeneity of NS, treatment with rhGH should be personalized and a conservative approach with judicious surveillance is recommended. Depending on the genotype, an individualized follow-up and close monitoring during rhGH treatments, also focusing on screening for neoplasms, should be considered.

## Introduction

Noonan syndrome (NS, OMIM PS163950) is one of the most common non-chromosomal disorders affecting development and growth (1). It is largely transmitted as a dominant trait, even though two recessive forms have recently been identified (2, 3). NS was first described by the cardiologist Jaqueline Noonan in 1968 (4), who reported a previously unrecognized phenotype with pulmonary valve stenosis (PVS), short stature, variable cognitive deficits, and facial dysmorphism as recurrent major features. NS is a syndromic condition characterized by a distinctive facial gestalt (*e.g.*, relative macrocephaly, hypertelorism, ptosis, and low-set/posteriorly rotated ears), failure to thrive in the first years of life, reduced postnatal growth, a wide spectrum of congenital and acquired cardiac defects (most commonly PVS and hypertrophic cardiomyopathy [HCM)]), varying degrees of developmental delay (DD)/intellectual disability (ID), typical chest deformities (superior pectus carinatum, inferior pectus excavatum), tendency to bleed and cryptorchidism in males (5–7).

**Figure 1:**
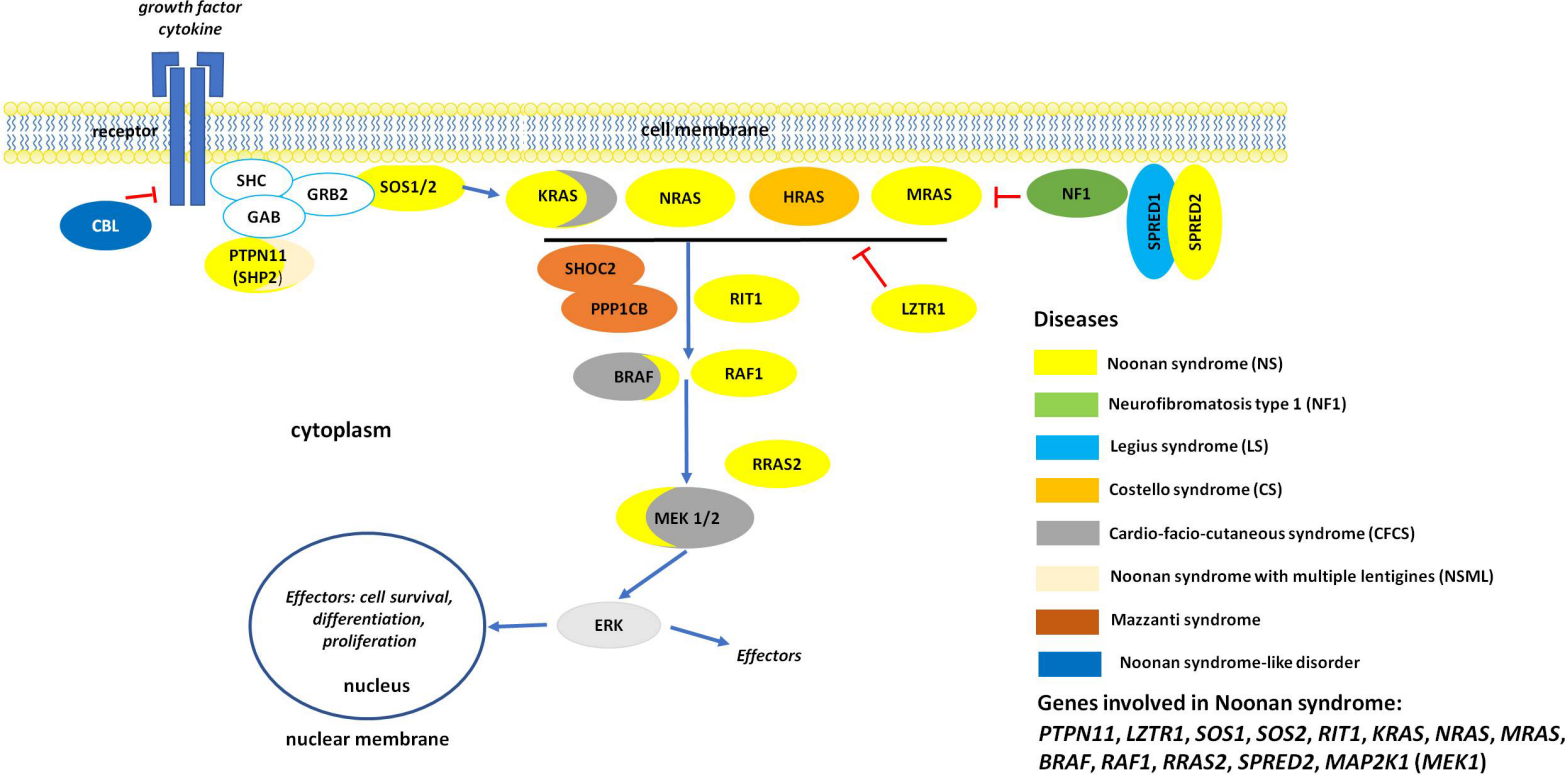
The RAS/MAPK signaling pathway and proteins involved in RASopaties. Overview of the RAS-MAPK signal transduction pathway. As shown, an extracellular stimulus triggers the activation of cell surface receptors (here a tyrosine kinase receptor, RTK), whose activation promotes the translocation of signal transducers positively controlling the function of RAS proteins (e.g., SOS1/2). These small monomeric GTPases, when activated, mediate the activation of the RAF kinases (BRAF and RAF1), which in turn phosphorylate and activate the dual-specificity kinases MEK1/2. Activated ERK1/2 is the last tier of the cascade and controls the function of a number of cytoplasmic and nuclear proteins. MAPK signaling is switched off by multiple circuits involving other proteins mutated in NS and related disorders (*e.g.*, neurofibromin, CBL, LZTR1, SPRED1 and SPRED2).

The variable clinical phenotype of NS overlaps with those of other genetic syndromes originally categorized as NS-spectrum disorders (NSSD) (**Figure 1**). Among these, NS with multiple lentigines (NSML, previously known as LEOPARD syndrome; OMIM 151100), Mazzanti syndrome (also known as NS-like disorder with loose anagen hair) (NS-LAH; OMIM 607721 and 617506), Legius syndrome (LGSS; OMIM 611431), and a phenotype originally denominated neurofibromatosis-NS (OMIM 601321), a condition representing a distinct form of neurofibromatosis 1 (NF1, OMIM 162200) are the most closely related conditions. There are also significant clinical overlaps with cardiofaciocutaneous syndrome (CFCS; OMIM 115150), and Costello syndrome (CS; OMIM 218040) (8–10), and other recently recognized diseases (11, 12). Unsurprisingly, the molecular mechanisms underlying the pathogenesis of these disorders are closely related. These disorders are caused by germline mutations in genes encoding components of the RAS-mitogen-activated protein kinase (MAPK) pathway, a signal transduction cascade controlling key cellular processes (*e.g.*, proliferation, differentiation, migration and metabolism) in response to a wide array of growth factors, hormones and cytokines (**Figure 1**) (13). Because of these shared mechanisms, these disorders are collectively termed as RASopathies (7–10). Multiple key players in this pathway are mutated in NSSD, with an overall gain-of-function effect on signal flow through the RAS-MAPK pathway (9).

The wide clinical variability characterizing NS results from a particularly marked genetic heterogeneity. Heterozygous germline mutations in 12 genes have been reported to be associated with NS (*PTPN11*, *SOS1*, *SOS2*, *KRAS*, *NRAS*, *RIT1*, *MRAS*, *RAF1*, *BRAF*, *MAP2K1*, *LZTR1*, and *RRAS2*) (1, 6, 8, 10, 14). Moreover, at least two genes have been reported as causing recessive forms of NS (*LZTR1* and *SPRED2*) (2, 3). Mutations in the *PTPN11* gene account for approximately 50% of NS cases, and mutations in *LZTR1*, *RIT1*, *SOS1* and *RAF1* for the majority of the remaining cases (1, 6–8, 10). Genetic mutations have not been identified in a small proportion of subjects with a clinical diagnosis of NS, supporting the existence of additional genes implicated in the disorder (14). Most NSSD causative pathogenic variants act through a gain of function mechanism on the RAS/MAPK signaling cascade that destabilizes the autoinhibitory mechanisms that maintain these proteins in their catalytically inactive conformation. Inactivating pathogenic variants affecting regulatory proteins that negatively control the RAS/MAPK cascade may also cause NSSD, and may act as either loss-of-function (LoF) (*e.g.*, *SPRED1*, *SPRED2*, neurofibromin and *LZTR1* in the recessive form of NS) or dominant-negative (DN) (*LZTR1* in the dominant form of NS) mutations (3, 11, 15, 16).

From an auxological point of view, NS is one of the most prevalent non-chromosomal disorders affecting development and growth (17, 18). In affected subjects, birth weight and length are generally normal, although postnatal growth failure is observed (19, 20). Commonly, height tends to follow the third centile from ages two to four years until puberty, when below-average height velocity and an attenuated pubertal growth spurt tend to occur with a near adult height in the lower limits of normal values (21–23).

In NS, decreased insulin-like growth factor I (IGF-I) and IGF binding protein 3 (IGFBP-3), together with low responses to GH pharmacological provocation tests (GHST), may suggest, for some patients, an impaired growth hormone (GH) release or disturbance of the GH/IGF-I axis. Variable GH resistance, particularly in subjects with *PTPN11* pathogenic variants, has also been reported (21). Short stature may be in part related to other factors (24, 25), such as congenital heart disease (CHD) requiring surgery (25); in fact, cardiac involvement is a main clinical feature (70-80%) of NS and cardiac anomalies are mostly represented by congenital heart diseases (in particular PVS) and HCM (20%) (25). Nearly 50% of NS patients present with some electrocardiographic abnormalities, even in the absence of structural cardiac abnormalities (25).

Emerging evidence shows that the molecular cause underlying the disease has a specific impact on stature, as demonstrated by a more severely impaired growth in patients carrying *PTPN11*, *RAF1*, and *KRAS* pathogenic variants compared to those with *SOS1* variants (24).

Although neurocutaneous manifestations have been considered hallmark features in NS and other RASopathies, other organs and systems, including the musculoskeletal system, may be affected (26). Musculoskeletal anomalies include scoliosis, kyphosis, anterior chest wall anomalies, pes planus, osteopenia, and hand anomalies (26, 27). The central nervous system may also be affected by congenital malformations; a few cases of Arnold-Chiari I malformation have been described, though the true incidence in NS is not known (28, 29).

Finally, NS is associated with a higher risk for benign and malignant proliferative disorders, such as juvenile myelomonocytic leukemia (JMML) and/or other hematological malignancies, as well as solid tumors, specifically neuroblastoma, solid brain tumors, and embryonal rhabdomyosarcoma (30–33).

NS diagnostic criteria were originally published by van der Burgt in 2007 (34). They are of particular value in research, and they were the basis for the more recently developed guidelines by Dyscerne (35), which set out recommended baseline investigations and age-specific management of patients. Similar recommendations are provided by Romano et al. (36), Roberts et al. (1) and, after GH treatment approvals in Europe, by Carcavilla et al. (8).

After 2007, when the Food and Drug Administration (FDA) approved recombinant human GH (rhGH) treatment for short stature in NS, the treatment was subsequently approved in Brazil, Israel, Japan, South Korea, Switzerland and some European countries (8, 17). For this reason, many studies conducted in the past evaluating the response and safety of rhGH treatment in NS patients were performed in subjects with concomitant GH deficiency (GHD) who were therefore treated with standard doses for this diagnosis. To date, most studies on rhGH treatment for NS have been retrospective and observational, involving small numbers of patients, with variable ages at the start of treatment and treated with different doses. Unfortunately, molecular characterization is available for only a small number of these cohorts (8, 17).

Here, we review and critically assess data from the literature on the growth pattern characterizing NS, altered GH/IGF-1 axis, and efficacy and safety of rhGH treatment in NS. PubMed and Google scholar tools were used to retrieve relevant publications by using the following terms: Noonan syndrome, *PTPN11*, *KRAS*, *SOS1*, *RAF1*, *NRAS*, *BRAF*, *MEK1*, *RIT1*, *SOS2*, *LZTR1*, *MRAS*, *CBL*, *RRAS*, *RRAS2, RASA2*, *SPRED1*, growth, growth hormone, GH, GH treatment, puberty, scoliosis, cancer, tumor, brain tumor, MRI, Chiari malformation, dysembryoplastic, pilocytic, medulloblastoma, oligodendroglioma, glioneuronal, astrocytoma, glioma, ependymoma,pulmonary stenosis, and hypertrophic cardiopathy. We included reviews, case reports, case series and case report abstracts.

## Growth and growth hormone-IGF-1 axis in Noonan syndrome

Up to 70% of NS subjects present with postnatal short stature (17, 37), even though the majority has a normal birth weight and length (20); the presence of edema may result in an overestimation of weight which should be carefully evaluated (20). Some authors have reported a significantly higher frequency of NS subjects who are small for gestational age (SGA) compared with the general population (24%), particularly for length (24), and in specific genotypes (24, 38). Attention should be paid to SGA patients whose phenotypic characteristics are suggestive of a RASopathy, and to children who do not respond to GH treatment (39). The concomitant diagnosis of SGA and NS may affect the responsiveness of a child to the growth-promoting effects of rhGH treatment (39). Prematurity has also been reported in a significant proportion of patients and should be taken into account when evaluating NS individuals (24, 40).

Feeding difficulties in NS subjects are extremely frequent (19) and may cause transient failure to thrive and poor weight gain during the neonatal period and infancy in 55 to 100% of cases, depending on the molecular subtype (20, 41).

Birth length is, however, usually normal, although postnatal growth failure is commonly observed starting during the first years of life (20); in fact, mean height tends to follow the third centile from ages two to four years until puberty, when below-average height velocity and an attenuated pubertal growth spurt tend to occur (20).

As bone age is usually delayed, prolonged growth into the 20s may occur, attenuating the growth deficit in some subjects (20). Near adult height approaches the lower limits of normal values: 161-167 cm in males and 150-155 cm in females, with a -1.5 SDS compared to the normal population (20, 21). On the contrary, more than 50% of females and nearly 40% of males have an adult height below the third centile (22).

Growth charts, not genotype-specific, have been developed, although evidence for the occurrence of short stature is represented by pooled data expressed in standard deviation scores (SDS) from patients at different ages, thus precluding a longitudinal description of growth (20–22). Currently available NS-specific growth charts should be used with caution, as they are not genotype-specific and often refer to patients for whom the clinical diagnosis had not been molecularly confirmed.

As reported, in patients with NSSD, the underlying molecular cause of the disease has a specific impact on stature (24). In fact, growth retardation appears to be significantly less severe and less frequent in patients with NSML and NS associated with *SOS1* mutations compared to patients with NSSD associated with pathogenic variants in other RASopathy genes, such as patients with *PTPN11*, *RAF1*, and *KRAS* mutations (24).

NS individuals generally present with a normal BMI during the first years of life (24, 42), followed by relative ‘thinness’ with a BMI in the lower normal range; it is rare for NS patients to be overweight or obese, suggesting that NS-causing mutations could impact energy metabolism regulation (36, 43, 44). The greatest decrease in BMI has been associated with pathogenic *SHOC2, KRAS* and *HRAS* variants, occurring in NS-LAH, CFCS and CS. These patients present with marked failure to thrive substantially due to muscle tissue wasting rather a decrease in adipose tissue (44). A possible correlation between metabolism and control of energy storage has been hypothesized (45, 46), which may involve two important hormones involved in unsatiety signals (insulin and leptin) and the RAS/MAPK pathway. As proof, patients with CS display an increased resting energy expenditure and a high calorie intake compared with the recommended levels of energy intake (47).

There may be other metabolic alterations in RASopathies. In the NSML mouse model, reduced fat mass and resistance to diet-induced obesity with increased carbohydrate metabolism/insulin sensitivity has been reported (48). On the other hand, the NS mouse model shows an insulin resistant phenotype associated with inflammation of tissues involved in the regulation of glucose metabolism likely due to increased activation of macrophage and triggered monocyte infiltration through SHP2 induced RAS/MAPK hyperactivation (49).

Puberty is often delayed in NS subjects (5), in both females (22%) and males (34%) (50). Those with delayed puberty, frequently resembling a constitutional delay of growth and puberty, are usually shorter and thinner than NS individuals with normal puberty (50). Unfortunately, the molecular available data do not allow us to make accurate genotype-phenotype correlations (50).

As NS is a condition characterized by clinical variability and genetic heterogeneity, different mechanisms implicated with altered GH secretory dynamics or response have been reported, including GHD, neurosecretory dysfunction, and GH insensitivity (GHI) (17, 21). Decreases in IGF-I and IGF-binding protein 3, together with low responses to provocation tests, suggest impaired GH release, or disturbance of the GH/IGF-I axis, at least in a proportion of affected individuals (21).

Recent data suggest that germline activating *BRAF* mutations may lead to an abnormal differentiation of pituitary hormone-producing cells in the progenitors of the pituitary gland with postnatal hypopituitarism, suggesting a biological role of the MAPK pathway in the etiology of pituitary hormone deficiencies, and a biological link between congenital forms of human hypopituitarism and RASopathies (51). Data on hypopituitarism in NS are poor (52) and there remain many questions about the presence of GHD in subjects with NS.

In NS with growth impairment, the results of provocatory tests should also be taken into account as data indicate that approximately 40% of NS subjects have a GH peak below 10 ng/mL after GHST (53, 54) and some individuals may also present with a range of disturbances in GH secretion, such as low levels of mean overnight GH concentration and unusual GH pulsatility with high trough GH concentration, indicating a concurrent presence of a neurosecretory dysfunction (55). On the other hand, a recent paper studying 24hGH profiles in patients with NS or Turner syndrome (TS), and unaffected prepubertal children showed that GH-baseline, GH mean values, GHmax and other parameters were significantly higher in NS patients, particularly in those with *PTPN11* variants, compared to healthy children (56). In fact, a mild GHI, related to a post-receptor signaling defect due to upregulation of SHP2, the protein encoded by *PTPN11*, has been reported in NS individuals with pathogenic *PTPN11* variants (21). SHP2 acts as a negative regulator of the GH receptor signaling pathway and its anomalous activation could be partially compensated *via* a more elevated GH secretion, as occasionally observed in NS subjects treated with rhGH who showed a mild resistance to rhGH treatment (21). NS individuals carrying *PTPN11* variants also have lower levels of insulin-like growth factor1 (IGF-1) than those without a *PTPN11* variant (37) although it is not completely clear whether this ​​ indicates GHD or GHI in these individuals. We believe that it is important to assess whether sensitivity to GH is normal or decreased before introducing rhGH treatment (57). In a short and/or slowly growing NS child, serum IGF-I levels could be useful in distinguishing between GHD or GHI (57). If serum IGF-I is low (or in the lower half of the reference range) for age, sex, and pubertal stage, or height velocity is reduced, assessment of the endogenous GH reserve by GHST could be informative (58). “Classical” GHD can be excluded in the presence of a normal stimulated GH peak (57). If a diagnosis of GHD is established, we suggest that magnetic resonance imaging (MRI) of the hypothalamic/pituitary region is also carried out (58).

## Effectiveness of growth hormone treatment

After 2007, when the use of rhGH received approval for treatment of short stature in NS by FDA, many countries (*e.g.*, Brazil, Israel, Japan, South Korea, and some European countries), began to treat NS patients (59–64). In addition to cases treated for documented GHD, rhGH treatment has also been initiated in NS individuals in the presence of markedly short stature, defective function of the GH-IGF-I axis, and/or a documented response to rhGH treatment (59–62). No standard dose has been established, however, based on available data from Phase III clinical trials (65, 66), another long-term interventional study (61) and long-term real world data from the NordiNET IOS and ANSWER international registries (67), the recommended dose is 0.066 mg/kg/day (0.46 mg/kg/week); however, we suggest an initial dose regimen of 0.033 mg/kg/day which may be increased up to 0.066 mg/kg/day in cases of poor response, as reported on the medication label and also stated by other authors (8).

Unfortunately, it is difficult to systematically compare published data on rhGH treatment in NS due to heterogeneous protocols, potential enrolment bias, the small size of the cohorts studied, different cohort selection criteria and varying durations of therapy (**Table 1**) (18, 54, 85). Most data on response to rhGH treatment derive from uncontrolled observational studies, frequently involving small numbers of patients, with different ages at onset of therapy, different rhGH doses, and varying durations of treatment (18). Noticeably, most individuals treated with rhGH were not genetically characterized, which is a drawback due to the marked clinical variability characterizing the disorder and reported genotype-phenotype correlations.

**Table 1:** Studies assessing the use of rhGH in Noonan syndrome.

Design	Patients (M:F)	GeneticTest	Age at start(years)	Duration(years)	rhGH dose (mg/kg/day)	Main concerns	Authors and references	
Retrospective longitudinal	6 (3 M, 3 F)	No genetic data	8.5 to 12.8	1.0	0.033	Small study; short follow-up; only clinic diagnosis	Ahmed ML et al., 1991	(68)
Retrospective longitudinal	5 (4 M, 1 F)	No genetic data	3.9 (2.5 - 6.0)	2.9	0.050	Small study; only clinic diagnosis	Thomas BC et al., 1993	(69)
Retrospective longitudinal	4 (4 F)	No genetic data	8.3 – 11.1	3.0 or 4.0	0.028	Small study; only clinic diagnosis	Municchi G et al., 1995	(42)
Uncontrolled trial	30 (19 M, 11 F)	No genetic data	8.9 ± 0.5	1.0	0.047	Only clinic diagnosis; short follow-up	Cotterill AM et al., 1996	(70)
Uncontrolled trial	23 (18 M, 5 F)	No genetic data	9.4 ± 3.0	1.0	0.052	Only clinic diagnosis; short follow-up	de Schepper J et al., 1997	(71)
Uncontrolled trial	12 (3 M, 9 F)	No genetic data	8.0 ± 4.1	1.0	0.040	Small study; only clinic diagnosis; short follow-up	Soliman AT et al., 1998	(72)
KIGS (observational)	66 (54 M, 12 F)	No genetic data	10.2 ± 3.3	5.3	0.037	Only clinic diagnosis; no genetic data available	Kirk JM et al., 2001	(73)
Uncontrolled trial	23 (16 M, 7 F)	No genetic data	9.3 ± 2.6	3.0	0.047	Only clinic diagnosis; no genetic data available	Macfarlane CE et al., 2001	(74)
Uncontrolled trial	14 (8 M, 6 F)	No genetic data	7.5 ± 2.5	2.0	0.026	Small study; short follow-up; only clinic diagnosis	Ogawa M et al., 2004	(59)
Uncontrolled trial	14 (10 M, 4 F)	*PTPN11* (7); no mut (7)	12.3 ± 3.5	3.0	0.047	Small study; only half patients with genetic data	Ferreira LV et al., 2005	(75)
Uncontrolled trial	10 (4 M, 6 F) 15 (8 M, 7 F)	No genetic data	7.5	7.7	0.033 0.066	Only clinic diagnosis; no genetic data available	Osio D et al., 2005	(61)
Uncontrolled trial	29 (19 M, 10 F)	*PTPN11* (16); no genetic data (11)	7.4 ± 2.2 6.3 ± 1.9	1.0	0.042 (mut) 0.050 (no mut)	Differences in IGF-I levels, GH peak between *PTPN11* mut and no mut; short follow-up	Binder G et al., 2005	(21)
Uncontrolled trial	35 (19 M, 16 F)	*PTPN11* (19); no genetic data (16)	10.4 ± 3.1	2.0	0.043 (prepub) 0.066 (pubertal)	short follow-up; only clinic diagnosis in the half of patients	Limal JM et al., 2006	(76)
KIGS (observational)	402 (242 M, 118 F)	No genetic data	9.73	3.0 (73 pts)	0.034	Only clinic diagnosis; high ceased treatments	Raaijmakers R et al., 2008	(77)
Controlled trial	29 (21 M, 8 F)	*PTPN11* (22); *SOS1* (1); *BRAF* (1); no mut (5)	11	6.4	0.050	–	Noordam C et al., 2008	(60)
NCGS (observational)	252 (174 M, 78 F)	No genetic data	9.8 ± 3.6	5.0 ± 2.6	0.045	Only clinic diagnosis	Romano AA et al., 2009	(62)
Observational study	19 (14 M, 5 F)	*PTPN11* (10); *SOS1* (2); *KRAS* (1); *MEK1* (1); no mut (5)	8.3 ± 2.4	1.0	0.066	Excluded pituitary hormone deficiencies; hypertrophic cardiomyopathy; short follow-up	Choi JH et al., 2012	(78)
Retrospective longitudinal	78 (47 M, 41 F); 33 treated for GHD	*PTPN11* (23); *RAF1* (1); *KRAS* (1); *BRAF* (1); *SHOC2* (7)	6.9 ± 3.6	9.3 ± 4.0	0.035	–	Tamburrino F et al., 2015	(54)
Retrospective longitudinal	5 (2 M, 3 F)	*PTPN11* (4); *KRAS* (1)	8.5 ± 3.1	5.0	0.033	Small study; only NS with GHD	Zavras N et al., 2015	(64)
NordiNet^®^ (observational) ANSWER (observational)	30 (24 M, 6 F)	No genetic data	8.4 ± 3.4	4.0	0.047 ± 0.010	Only clinic diagnosis	Lee PA et al., 2015	(63)
Retrospective longitudinal	124 (84 M, 40 F)	*PTPN11* (39); *SOS1* (1)	8.4 ± 4.5	3.0	0.035 ± 0.007	Poor genetic data; difference in height between GH treated and not treated	SŞiklar Z et al., 2016	(79)
Retrospective longitudinal	23 (14 M, 9 F)	*PTPN11* (7); *RAF1* (3); *SOS1* (2); No mut (11)	5.8 ± 2.6	3.0	0.060	11 patients without mutations	Jo KJ et al., 2019	(80)
Retrospective longitudinal	42 (29 M, 13 F)	*PTPN11* (35); *RAF1* (3), KRAS (2), *SOS1* (1), *SHOC2* (1)	11.4 ± 3.4	5.1 ± 2.0	0.033 - 00.66	Patients with chronic cardiopathies excluded; many patients discontinued the treatment	Malaquias AC et al., 2019	(81)
KIGS (observational)	613 (389 M, 224 F)	*PTPN11* (19.9%); No mut (491)	9.60	Near 5.0	0.037	Only *PTPN11* mutations; no other genetic data	Ranke MB et al., 2019	(82)
NordiNet^®^ (observational) ANSWER (observational)	412 (292 M, 120 F) 84 (67 M, 17 F) EAS	Genetic data in 15.3%	8.4 ± 3.6	≥4 years	0.042	Only 15.3% of patients with genetic data available; difficult in evaluation data	Rohrer TR et al., 2020	(67)
Randomized, double-blind trial	25 (14 M, 11 F) 26 (18 M, 8 F)	*PTPN11* (28), *SOS1* (2), *KRAS* (1), *RAF1* (2), *BRAF* (1), *SHOC2* (1), *RIT1* (1)	6.6 ± 2.4 6.1 ± 2.2	4.0	0.033 0.066	Genetic data in 70.6%; uneven distribution of genotypes in the 2 groups	Horikawa R et al., 2020	(66)
Retrospective longitudinal	12 (10 M, 2 F)	No	8.0	1 - 8	0.037	Small study; only clinic diagnosis (no genetic data)	Apperley LJ et al., 2020	(83)
Retrospective longitudinal	228 (132 M, 96 F); 68 (40 M, 28 F) with GHD	*PTPN11* (48), *SOS1* (3), *KRAS* (4), *RAF1* (2), *BRAF* (2), *SHOC2* (9)	7.2 ± 3.5	6.3 ± 4.0	0.034	All subjects with genetic data; only GHD treated	Libraro A et al., 2021	(84)

Short and long term studies have demonstrated a consistent and significant increase in HV in NS children and adolescents treated with rhGH (57, 59–61, 67, 86). Increases in height SDS vary from 0.6 to 1.8 and may depend on age at start of treatment and duration of treatment, age at onset of puberty and/or GH sensitivity (59, 60, 86).

Many retrospective, observational studies on rhGH treatment in NS patients with and without GHD as well as clinical trials in NS patients with short stature have been carried out over the past 20 years (42, 54, 59, 60, 64, 68–72, 74, 75, 78–80, 83). In some studies, auxological and safety data were consequent to rhGH dosages established on the whole cohort of NS patients with no distinction being made between subjects with GHD and subjects without GHD, while other studies included only NS subjects with GHD (54, 59, 60, 64, 69, 72, 75, 78–80, 83). In other studies, doses of rhGH varied according to different parameters, for example the pubertal stage of patients (21, 61, 66, 76, 81). International Registers (*e.g.*, the Kabi International Growth Study [KIGS] database or the US National Cooperative Growth Study [NCGS]), report data for NS cohorts with and without GHD who were treated with rhGH with different dosages (62, 63, 67, 73, 77, 82).

Some short-term studies have demonstrated an increase in HV and an increase in mean height SDS (63, 68, 70, 71, 74), particularly in the first year of treatment (63, 68, 70, 71, 74), suggesting that short-term use of rhGH for managing short stature is effective in NS. In a study involving 30 patients with a clinical diagnosis of NS without genetic characterization, nearly 80% of patients (aged from 4.5 to 14.0 years and treated with 0.045 mg/kg/day of rhGH for 1 year) showed a significant increase in mean height SDS (-3.01 ± 0.1 to -2.36 ± 0.1) and HV (4.9 ± 0.2 to 8.1 ± 0.4 cm/yr) (70), highlighting the effectiveness of rhGH treatment in both prepubertal and pubertal patients (70, 76, 78). However, other studies indicate that high HV typically and gradually decreases every year after the first year of treatment (74, 78) despite adherence to therapy (63, 74). This waning effect is likely to be due to many co-occurring factors, such as age at the beginning of therapy, GH secretory status, and variable GHI. Indeed, a recent randomized, double-blind, multi-center trial investigating the effect of dose on the growth-promoting effect of rhGH in prepubertal children with NS demonstrated a significant increase in height gain with a dose of 0.066 versus 0.033 mg/kg/day (66). In addition to dose, factors associated with improved outcome include earlier initiation of rhGH therapy and longer prepubertal duration of therapy (61, 62, 82). The presence of genetic heterogeneity in NS also raises the possibility that different responses to rhGH treatment may be genotype-related (78). Unfortunately, genetic data are lacking in the majority of papers. Some data show that prepubertal NS children with GHD present an increased growth rate during rhGH treatment at a rate equivalent to girls with Turner syndrome but at a lower rate than in idiopathic GHD (63, 64). Nonetheless, these studies used very different rhGH dosages (63, 64).

Long-term data on the effect of rhGH treatment on height outcomes are poor and limited to a small number of patients. Data on adult height (AH) or near-adult height (NAH) in NS patients treated with rhGH are also available (42, 54, 61–63, 76, 77, 82, 87, 88), but refer to small cohorts whose results may be biased because of a number of factors, such as age at start of treatment, duration of treatment, and definitions of NAH (60–62, 81, 82).

Patients who were treated with rhGH for more than 3 years (median, 6.4 years) showed an increase in height of 1.3 SDS (26), with the majority of patients achieving an AH within the normal range and 30% remaining below -2 SDS (37, 84). Again, these differences might be due to several confounding factors (37), as well as the dose of rhGH (84). After one year of rhGH treatment, the mean difference between chronological age and bone age decreased (68), and this was particularly evident in subjects having a significantly delayed bone age at the start of rhGH therapy (62), reflecting a normalization rather than excessive acceleration of bone development in these individuals (37).

The bone age of rhGH-treated NS individuals with a significant increase in AH did not excessively advance during rhGH treatment (60, 74). Some data also show that, after rhGH treatment, most NS patients present with significant gains in AH, despite the pubertal growth spurt occurring much later than normal (37).

However, the possible relationship between a “reduced” advancement in bone age with later pubertal development and the effect on final stature is not clear.

Some studies report a more significant increment in height SDS after one to three years of rhGH treatment in patients without *PTPN11* mutations (75, 76, 87). However, other data do not confirm these findings (78).

There are contrasting data about the influence of pre-treatment values of IGF-1 and IGFBP-3 on the effectiveness of rhGH treatment. While some studies suggest that basal IGF-I and IGFBP-3 levels before rhGH treatment are significantly related to final response (60), other data indicate that these levels cannot predict changes in height SDS (63, 67). Other studies reported significantly lower IGF-1 and IGFBP-3 levels at the start of rhGH therapy in some NS individuals carrying *PTPN11* mutations (37). The choice of reference population is important when interpreting the magnitude of rhGH response. This is clearly illustrated by the differences in mean adjusted ΔHSDS at 5 years (national reference, 1.17, Ranke 1.46), though a similar effectiveness of treatment was observed irrespective of the reference used (88).

In summary, the available data confirm that rhGH treatment is associated with an increase in HSDS in NS individuals during childhood with a final increase in AH. It seems that the earlier rhGH treatment is started, the more likely an optimal height is reached due to height normalization and the delayed pubertal onset frequently observed in NS subjects. However, the scarcity of data on genetically characterized cohorts does not allow us to accurately determine whether response to rhGH treatment also depends on genotype.

## Overview of cautions and side effects during rhGH treatment

**Tables 2** and (**3A**, **3B** and **3C**) show the results of the major studies on rhGH therapy in NS. Side effects in children were infrequently reported. Based on the data, rhGH treatment does not seem to influence cardiac physiology and function (38). The decision to use rhGH in patients with NS should, however, be made on a case-by-case basis (8). The accumulated safety data on rhGH treatment in NS are reassuring (60) and include no significant evidence of adverse cardiac effects or increased occurrence of malignancies (60–62).

**Table 2:** Studies evaluating the course of cardiac anomalies during rhGH treatment in rasopathies.

Patients (M:F)	GeneticTest	Duration(years)	rhGH dose (mg/kg/day)	Pre-treatmentcardiac anomalies	cardiac anomalies course	Concerns		Authors and references	
4 (4 F)	No genetic data	3.0 or 4.0	0.023	No data	No progression	Only clinic diagnosis	Municchi G et al., 1995		(42)
30 (19 M, 11 F)	No genetic data	1.0	0.047	1 HCM (19 pts with cardiopathy)	No progression	Only clinic diagnosis; short follow-up	Cotterill AM et al., 1996		(70)
23 (18 M, 5 F)	No genetic data	1.0	0.052	No HCM (19 pts with cardiopathy)	No progression	Only clinic diagnosis; short follow-up; HCM no selected?	de Schepper J et al., 1997		(71)
12 (3 M, 9 F)	No genetic data	1.0	0.040	No HCM (7 pts with cardiopathy)	No data	Only clinic diagnosis; short follow-up; HCM no selected?	Soliman AT et al., 1998		(72)
66 (54 M, 12 F)	No genetic data	5.3	0.037	78% with cardiac malformations	1 decompensation (no data)	Only clinic diagnosis; echocardiograms performed in 86%	Kirk JM et al., 2001		(73)
23 (16 M, 7 F)	No genetic data	3.0	0.047	No HCM (12 pts with cardiopathy)	No HCM development	Only clinic diagnosis; light progression wall thickness in 2 pts	Macfarlane CE et al., 2001		(74)
27 (21 M, 6 F)	No genetic data	2.0 to 5.0	0.050	1 HCM (19 pts with cardiopathy)	No progression	Only clinic diagnosis	Noordam C et al., 2001		(89)
23 (16 M, 7 F)	No genetic data	3.0	0.047	No HCM (11 pts with cardiopathy)	No progression	Only clinic diagnosis	Brown DC et al., 2002		(90)
14 (8 M, 6 F)	No genetic data	2.0	0.024	No data; serious cardiac dysfunction excluded	No data	No genetic data; HCM no selected? short follow-up	Ogawa M et al., 2004		(59)
14 (10 M, 4 F)	*PTPN11* (7); no (PTPN11) mut. (7)	3.0	0.048 0.046	1 HCM (7 pts with cardiopathy)	HCM progression in one patient	Patients with HCM no *PTPN11*	Ferreira LV et al., 2005		(75)
29 (19 M, 10 F)	*PTPN11* (16); no genetic data (11)	1.0	0.042 (mut) 0.050 (no mut)	HCM: 2/16 *PTPN11*; 1/13 no genetic data;	No data	short follow-up	Binder G et al., 2005		(21)
35 (19 M, 16 F)	*PTPN11* (19); no genetic data (16)	2.0	0.043 (prepub) 0.066 (pubertal)	HCM patients excluded	No data	HCM patients excluded; short follow-up	Limal JM et al., 2006		(76)
402 (242 M, 118 F)	No genetic data	7.5	0.034	No data	No data	Do specific data are reported	Raaijmakers R et al., 2008		(77)
29 (21 M, 8 F)	*PTPN11* (22); *SOS1* (1); *BRAF* (1); no mut (5)	6.4	0.050	No data	No data	Cardiac dysfunctions excluded?	Noordam C et al., 2008		(60)
65 (35 M, 30 F)	No genetic data	5.0 ± 2.6	0.045	No data	Progression in two patients (one HCM)	Do specific and genetic data are reported	Romano AA et al., 2009		(62)
19 (14 M, 5 F)	*PTPN11* (10); *SOS1* (2); *KRAS* (1); *MEK1* (1); no mut (5)	1.0	0.066	79% with cardiac malformations; HCM patients excluded	No specific data	HCM patients excluded; short follow-up	Choi JH et al., 2012		(78)
78 (47 M, 41 F); 33 treated for GHD	*PTPN11* (23); *RAF1* (1); *KRAS* (1); *BRAF* (1); *HOC2* (7)	9.3 ± 4.0	0.035	76% had cardiac anomalies; HCM patients excluded?	No progression	HCM patients excluded?	Tamburrino F et al., 2015		(54)
5 (2 M, 3 F)	*PTPN11* (4); *KRAS* (1)	5.0	0.033	No data	Sure (no data)	No specific data	Zavras N et al., 2015		(64)
30 (24 M, 6 F)	No genetic data	4.0	0.047 ± 0.010	No data	No specific data	Only clinic diagnosis	Lee PA et al., 2015		(63)
124 (84 M, 40 F)	*PTPN11* (39); *SOS1* (1)	3.0	0.035 ± 0.007	62.9% with cardiovascular abnormalities	No progression	Only one third with genetic data	SŞiklar Z et al., 2016		(79)
23 (14 M, 9 F)	*PTPN11* (7); *RAF1* (1); *SOS* (1); No mut (7)	3.0	0.060	74% had congenital heart defects, 30% HCM	No progression	No specific data	Jo KJ et al., 2019		(80)
42 (29 M, 13 F)	*PTPN11* (35); *RAF1* (3), *KRAS* (2), *SOS1* (1), *SHOC2* (1)	5.1 ± 2.0	0.033 – 00.66	71% had cardiac defects; 3 patients HCM	Progression in 2 pts (*RAF1* and *SOS1*)	Chronic cardiopathies excluded? many treatments discontinued	Malaquias AC et al., 2019		(81)
140 (74 M, 66 F)	*PTPN11* (near 50%)	Near 5.0	0.037	No data	No specific data	Cardiac system problems in 4, left ventricular hypertrophy in 2	Ranke MB et al., 2019		(82)
412 (292 M, 120 F) 84 pts for EAS	*PTPN11* (56), *KRAS* (2), *SOS1* (2), *RAF1* (5), *SHOC2* (1)	≥4 years	0.042	Cardiac comorbidities likely under-reported at 8.3%	5 patients (no data); no HCM	Poor genetic data (only 15.3%)	Rohrer TR et al., 2020		(67)
25 (14 M, 11 F) 26 (18 M, 8 F)	70.6% (*PTPN11* 28, *SOS1* 2, *KRAS* 1, *RAF*1 2, *BRAF* 1, *SHOC2* 1, *RIT1* 1	4.29 4.16	0.033 0.066	3 patients with HCM (12.%) 5 patients with HCM (19.2%)	No progression	No specific data about HCM and genotypes	Horikawa R et al., 2020		(66)
12 (10 M, 2 F)	No genetic data	1 – 8	34	No data	No specific data	Only clinic diagnosis	Apperley LJ et al., 2020		(83)

EAS, effectiveness analysis set; HCM, hypertrophic cardiomyopathy.

**Table 3A:** Primary brain tumors in Noonan syndrome with PTPN11 mutations.

Gender	Age	Mutation	Tumor diagnosis	Location	Previous rhGH treatment (Y/N)	rhGH treatment (years)	GH deficiency (Y/N)	Authors	Ref
M	16	Clinical diagnosis	Pilocytic astrocytoma	Intramedullary spinal cord	Ukn	–	Ukn	Sanford RA et al., 1999	(91)
M	20	Clinical diagnosis	Glioma	Unknown	Ukn	–	no	Takagi M et al., 2000	(92)
F	18	*PTPN11* (p.Glu139Asp)	Oligodendroglioma	Hypothalamus	Ukn	–	Ukn	Jongmans M et al., 2005	(93)
Ukn	24	*PTPN11* (p.Thr22Ala)	Oligodendroglioma	Unknown	Ukn	–	Ukn	Martinelli S et al., 2006	(94)
F	11	Clinical diagnosis	Pilocytic astrocytoma	Sellar/suprasellar	Ukn	–	Ukn	Fryssira H et al., 2008	(95)
M	6	*PTPN11* (pAsn58Asp)	Low grade mixed glioneuronal tumor	Sellar/suprasellar and hypothalamus	Ukn	–	Ukn	Sherman CB et al., 2009	(96)
M	8	*PTPN11* (p.Pro491Phe)	Pilocytic astrocytoma	Sellar/suprasellar and prepontin	Ukn	–	Ukn	Schuettpelz LG et al., 2009	(97)
Ukn	Ukn	Clinical diagnosis?	Ukn	Left parietal lobe tumor	**Yes**	**Ukn**	**Ukn**	Romano AA et al., 2009	(62)
Ukn	13	Clinical diagnosis?	DNET	Left parietal lobe	**No**	**After diagnosis**	**No**	Selter M et al., 2010*	(98)
Ukn	10	*PTPN11* (p.Gly60Ala)	DNET	Temporal lobe	Ukn	–	Ukn	Jongmans MCJ et al., 2011	(30)
M	21	*PTPN11* mutation	Multiple indeterminate lesions (MRI)	Supratentorial, infratentorial, cortical and thalamus	**Yes**	**Ukn**	**No**	De Jong M et al., 2011	(99)
M	18	Clinical diagnosis	Glioneuronal tumor	Fourth ventricle	Ukn	–	Ukn	Karafin M et al., 2011		(100)
M	17	*PTPN11* (p.Asn58Lys)	DNET	Occipital cortex	Ukn	–	Ukn	Bendel A et al., 2012	(101)
M	37	Maternal uncle case above	DNET	Unknown	Ukn	–	Ukn	Bendel A et al., 2012	(101)
M	10	*PTPN11* (p.Thr468Met)	Medulloblastoma	Cerebellum	**No**	**After diagnosis**	**Yes (after)**	Rankin J et al., 2013	(102)
M	13	*PTPN11* (exon 3)	DNET	Left parietal lobe	**Yes**	**Ukn**	**Ukn**	Pellegrin MC et al., 2014*	(103)
M	13	*PTPN11*	DNET	Right parietal-occipital cortex	**Yes**	**Ukn**	**Ukn**	Pellegrin MC et al., 2014	(103)
M	12	*PTPN11*	DNET	Temporal lobe and thalamus	Ukn	–	Ukn	Delisle MB et al., 2014	(104)
M	9	*PTPN11* (p.Asp61Gly)	DNET	Temporal lobe and cerebellum	**Yes**	**15 months**	**No**	Krishna KB et al., 2014	(105)
M	16	*PTPN11* (p.Asn308Asp)	Pilocytic astrocytoma,	Fourth ventricle and	**Yes**	**13 months**	**No**	Krishna KB et al., 2014	(105)
				left lateral ventricle					
M	Ukn	*PTPN11* mutation	Low grade astrocytoma	Suprasellar and thalamic region	Ukn	–	Ukn	Rush S et al., 2014^	(106)
M	Ukn	*PTPN11* mutation	Low grade astrocytoma	Suprasellar and thalamic region	Ukn	–	Ukn	Rush S et al., 2014^	(106)
M	Ukn	*PTPN11* mutation	Low grade astrocytoma	Suprasellar and thalamic region	Ukn	–	Ukn	Rush S et al., 2014^	(106)
F	14	*PTPN11* (p.Asn308Asp)	Hight grade glioma	Left brainstem/cerebellum	Ukn	**-**	Ukn	Bendel A, Pond D. 2014	(107)
F	7	*PTPN11* (p.Gly60Ala)	Pilocytic astrocytoma	Right optic nerve	Ukn	–	Ukn	Kratz CP et al., 2015	(108)
M	14	*PTPN11* (p.Glu139Asp)	Pilomyxoid astrocytoma	Right optic nerve	Ukn	–	Ukn	Nair S et al., 2015	(109)
M	8	*PTPN11* (p.Glu139Asp)	DNET	Temporal lobe and cerebellum	**Yes**	**4 years**	**No**	McWilliams GD et al., 2015	(110)
F	6	*PTPN11* (p.Asn308Asp)	DNET	Unknown	Ukn	–	Ukn	Kratz CP et al., 2015	(108)
M	16	*PTPN11* (p.Asn308Asp)	DNET	Left temporal and frontal lobe, right thalamus	**No**	–	Ukn	Siegfried A et al., 2016	(111)
F	14	*PTPN11* (p.Asn308Asp)	Anaplastic astrocytoma	Left brainstem/cerebellum	Ukn	–	Ukn	El-Ayadi M et al., 2019	(112)
M	9	*PTPN11* (p.Thr2lle)	Anaplastic astrocytoma	Third ventricle	Ukn	–	Ukn	El-Ayadi M et al., 2019	(112)
M	11	*PTPN11* (p.Asn308Asp)	Subependymoma	Intraventricular mass	**Yes**	**6 years**	**Yes**	Boonyawat B et al., 2019	(113)
F	9	*PTPN11* (p.Asn308Asp)	Pilocytic astrocitoma and glioneuronal tumor	Cerebellum and right temporal lobe	**No**	–	Ukn	Lodi M et al., 2020	(31)
M	15	*PTPN11*	Ukn	Brain neoplasm and metastases to the spine	**Yes**	**Ukn**	**Ukn**	Rohrer TR et al., 2020	(67)
F	9	Ukn	Glioneuronal tumor	Ukn	**Yes**	**Ukn**	**Ukn**	Rohrer TR et al., 2020	(67)
M	9	Ukn	Ukn	Brain neoplasm	**Yes**	**Ukn**	**Ukn**	Rohrer TR et al., 2020	(67)
M	27	*PTPN11* (p.Asn308Asp)	Ganglioneuroma	Paravertebral	**Yes**	**Ukn**	**Yes**	Morales-Rosado JA et al., 2021	(114)
F	12	*PTPN11*	Glioblastoma multiforme	Thoracolumbar spine	**No**	**-**	**Ukn**	Khan A et al., 2021	(115)

DNET, dysembryoplstic neuroepitelial tumor.

**Table 3B:** Other (no hematological) primary tumors in Noonan syndrome with PTPN11 mutations.

Gender	Age	Mutation	Tumor diagnosis	Location	Previous rhGH treatment (Y/N)	GH deficiency (Y/N)	Authors	Ref
M	0.5	*PTPN11* (p.Gly60Ala)	Neuroblastoma	Mediastinal and retroperitoneal	No	No	Mutesa L et al., 2008	(116)
Ukn	0.1	*PTPN11* (p.Asn308Asp)	Hepatoblastoma	Abdomen	No	No	Yoshida R et al., 2008	(117)
Ukn	1	*PTPN11* (p.Ile282Val)	Neuroblastoma	Adrenal gland	No	No	Jongmans MCJ et al., 2011	(30)
M	0.6	*PTPN11* (p.Ser502Thr)	Neuroblastoma	Left-sided adrenal gland	**No**	Ukn	Kondoh T et al., 2011	(118)
F	4	*PTPN11* (p.Asn308Asp)	Neuroblastoma	Mediastinal and right adrenal	**No**	Ukn	Chantrain CF et al., 2007	(119)
F	3	*PTPN11* (p.Ile282Met)	Neuroblastoma	–	**Ukn**	Ukn	Kratz CP et al., 2015	(108)
–	–	*PTPN11* (p.Asn308Asp)	Neuroblastoma	Mediastinum	Ukn	Ukn	Li X et al., 2019	(120)
M	6	*PTPN11* (no mut. reported)	Granular cell tumor	Scrotum	Ukn	Ukn	Sidwell RU et al., 2008	(121)
M	8	*PTPN11* (p.Asn308Asp)	Granular cell tumor	Skin (multiple sites)	Ukn	Ukn	Ramaswamy PV et al., 2010	(122)
F	10	*PTPN11* (no mut. reported)	Granular cell tumor	Skin (left forearm)	**Yes**	**No**	Moos D et al., 2012	(123)
M	12	*PTPN11* (p.Gly503Arg)	Granular cell tumor	Skin (left arm), tongue, lower lip	**Yes**	**No**	Bamps S et al., 2013	(124)
F	29	*PTPN11* (Thr468Met)	Granular cell tumor	Skin (buttock)	No	No	Park SH & Lee SH 2017	(125)
F	0.1	*PTPN11* (p.Gly409Ala)	Neuroblastoma	Spine and paravertebral thorax	No	No	Ekvall S et al., 2011	(126)
		*SHOC2* (p.Ser2Gly)						

**Table 3C:** Primary brain and other tumors in Noonan syndrome and other rasopathies with no PTPN11 mutations.

Gender	Age	Mutation	Tumor diagnosis	Location	Previous rhGH treatment (Y/N)	GH deficiency (Y/N)	Authors	
F	22	*LZTR1* (p.Arg284Cys)	Oligo-astrocytoma	Right fronto-temporo-insular	**Yes**	**No**	Jacquinet A et al., 2019	(127)
F	2	*SOS1* (p.Ser548Arg)	Embryonal rhabdomyosarcoma	Biliary ampulla/duodenum	No	Ukn	Hastings R et al., 2010	(128)
M	4	*SOS1* (p.Pro102Arg)	Embryonal rhabdomyosarcoma	Urachus	**Yes**	**Ukn**	Denayer E et al., 2010	(129)
M	4	*SOS1* (p.Met269Thr)	Sertoli cell tumor	Testis	**Yes**	**Ukn**	Denayer E et al., 2010	(129)
M	12	*SOS1* (p.Leu569Val)	Granular cell tumors	Skin	No	Ukn	Denayer E et al., 2010	(129)
M	4	*SOS1* (p.Lys728Ile)	Embryonal (botryoid)	Bladder	Ukn	Ukn	Jongmans MCJ et al., 2010	(93)
F	9	*SOS1* (p.Arg552Lys)	Unspecified Tumor	Brain	Ukn	Ukn	Abdelmoula NB et al., 2020	(130)
M	2	*KRAS* (p.Asp153Val)	Astrocytoma	Brain	Ukn	Ukn	Kratz CP et al., 2015	(108)
F	6	*NRAS* (p.Gly12Arg)	Embryonal rhabdomyosarcoma	Right thumb	No	No	Garren B et al., 2019	(131)
–	–	*HRAS*	Embryonal rhabdomyosarcoma	–	Ukn	Ukn	Aoki Y et al., 2005	(132)
F	9	*HRAS* (p.Gly12Ser)	Embryonal rhabdomyosarcoma	–	**Yes**	**Yes**	Gripp KW et al., 2005	(133)
M	2	*HRAS* (p.Gly12Ser)	Embryonal rhabdomyosarcoma		**No**	**Yes**	Gripp KW et al., 2005	(133)
F	21	*HRAS* (p.Gly12Ala)	Transitional cell carcinoma	Bladder	**Yes**	**Yes**	Gripp KW et al., 2005	(133)
–	7	*HRAS* (p.Gly12Ser)	Embryonal rhabdomyosarcoma	–	Ukn	Ukn	Kerr B et al., 2006	(134)
–	0.7	*HRAS* (p.Gly12Ser)	Embryonal rhabdomyosarcoma	–	Ukn	Ukn	Kerr B et al., 2006	(134)
–	5	*HRAS* (p.Gly12Asp)	Embryonal rhabdomyosarcoma	–	Ukn	Ukn	Kerr B et al., 2006	(134)
–	10	*HRAS* (p.Gly12Ser)	Embryonal rhabdomyosarcoma	–	** **	** **	Kerr B et al., 2006	(134)
M	2	*CBL* (p.Gln367Pro)	Embryonal rhabdomyosarcoma	Abdomen	No	No	Ji J et al., 2019	(135)
M	11	*RAF1* (p.Gly361Ala)	Glioma	Leptomeningeal	No	Ukn	Harms FL et al., 2017	(136)
F	0.9	*NRAS* (Gly12Arg)	Unspecified expansive lesion	Hypothalamus	No	No	Altmüller F et al., 2017	(137)
F	12	*CBL* (p.Gln367Pro)	Teratoma	Abdomen	No	No	Hanson HL et al., 2014	(138)
M	4	*RIT1* (p.Phe82Leu)	Tumor ndd	Testis	Ukn	Ukn	Yaoita M et al., 2016	(139)

We review some of the major concerns and side effects during rhGH treatment in NS individuals.

### 1) Metabolic profile and GH treatment

Several studies show normal blood glucose levels during rhGH treatment (37). Recently, the metabolic impact of SHP2 hyperactivation has been investigated in 21 NS children carrying *PTPN11* pathogenic variants. Although they presented with a lower BMI compared to normal weight healthy control subjects, they showed persistent increased glycemia and insulinemia levels after oral glucose tolerance testing (OGTT) (140). Such insulin resistance with reduced adiposity occurs without obvious signs of ectopic lipid deposits or lipotoxicity. This anomalous response is probably induced by a proinflammatory phenotype triggered by SHP2 hyperactivation which may alter hepatic macrophage homeostasis and promote insulin resistance. Larger studies are needed to further confirm this data (140).

Additional factors to be taken into account include height, age (early initiation maximizes prepubertal linear growth) and the presence of comorbidities. Nutrition should be assessed, and energy intake deficits resolved before initiation of treatment, and in cases of clinical features compatible with GHD, evaluation of the somatotropic axis should be considered.

Special attention should be paid to IGF-1 levels, carbohydrate metabolism and other possible adverse events. To the best of our knowledge, no specific studies on GH therapy at different dosages in patients with altered metabolic profiles have been performed.

If the patient exhibits a poor response despite 1-2 years of treatment at high doses, discontinuation of treatment should be considered, as the peak response is expected to occur in the early years of the treatment (8).

Patients with a clinical NS diagnosis without an identified molecular cause in known RASopathy genes should be considered for treatment with extreme caution; in these cases, assessment by an experienced clinical geneticist is recommended, as well as careful monitoring of the patient throughout treatment (8).

### 2) Cardiac anomalies and GH treatment

Cardiac involvement is one of the main clinical features of NS, occurring in at least 70-80% of individuals. The most common manifestations are congenital heart diseases (in particular PVS, 60-70% of patients) and HCM (nearly 20% of patients) (25). However, a wide spectrum of other abnormalities has been reported, including atrial and/or ventricular septal defects, pulmonary artery branch stenosis, and mitral valve or coronary artery anomalies (25).

Electrocardiographic abnormalities, such as multifocal atrial tachycardia, wide QRS intervals with a predominantly negative pattern in the left precordial leads and left axis deviation with giant Q waves, have also been reported in 50% of NS patients, even in the absence of structural cardiac abnormalities (25).

RAS signaling has a central role in both pathologic and physiologic cardiac hypertrophy as demonstrated in multiple *in vitro* and *in vivo* settings (141, 142). Expression of the dominant negative Raf-1 variant in mice has no effect on cardiac function at baseline but promotes cardiomyocyte apoptosis and increases mortality in settings of pressure overload (143).

Nonetheless, many issues, including the specific pathways activated by RAS GTPases which eventually lead to cardiac hyperplasia or hypertrophy, have not yet been elucidated (144). Among the RASopathies, the frequency of HCM is strictly correlated with the genotype, being particularly frequent in NS patients with *RAF1*, *LZTR1*, *RIT1* and *MRAS*, NSML (narrow spectrum of *PTPN11* mutations), and CS (narrow spectrum of *HRAS* mutations) (145–151).

Despite the good safety profile of rhGH treatment in NS patients presenting with HCM, a few adverse effects have been reported (**Table 2**). In a retrospective analysis (152), one case of HCM and one case of worsening HCM were reported (81, 152), but the genotype of the affected individuals is not known.

A progression of HCM associated with rhGH treatment has been reported in CS patients; nearly 20% of patients presented an increased severity of HCM during rhGH treatment (153). There is evidence of mild progression of PVS in NS individuals, but this does not appear to be related to rhGH treatment (60) (**Table 2**).

Two prospective studies specifically designed to evaluate cardiac anatomy and function after 1 and 4 years of rhGH therapy at different dosages, did not identify any change in myocardial function, or in ventricular wall thickness. Unfortunately, no genotype information on the enrolled patients were available (74, 89, 90).

An electrophysiologic phenotype has been also described in NSSD, with an increased incidence of multifocal atrial tachycardia and ectopic atrial tachycardia that occurs independently of HCM or PVS in 36% of patients (154–156). Calcium dysregulation may result in triggered activity giving rise to the atrial tachycardia, as well as contributing to the cardiomyopathy phenotype (154).

Unrelated arrhythmias have been described during rhGH treatment in NS (146, 147) some of which were evident also after interruption of the treatment (65, 157). This finding may be not strictly related to NS.

In conclusion, rhGH should be introduced only after a thorough cardiologic evaluation, particularly in patients carrying variants of specific genes (see previous paragraphs). NS patients undergoing rhGH therapy should always be closely monitored. At the first signs of HCM, discontinuing rhGH should be considered (150, 153). An assessment of the relative risks and benefits of rhGH treatment should be made for individual patients. Unfortunately, genotype-phenotype correlations are lacking from the vast majority of studies; we stress the need for collecting more complete data and longer follow-up

### 3) Cancer risk and GH treatment

Dysregulation of the RAS/MAPK signaling pathway may increase risks for cancer and contribute to oncogenesis (9, 30, 31, 158, 159). NS is associated with a higher risk for benign and malignant proliferative disorders, such as juvenile myelomonocytic leukemia (JMML) and other hematological malignancies, as well as solid tumors, specifically neuroblastoma, brain tumors, and embryonal rhabdomyosarcoma (30, 31, 105, 108, 110, 112, 127, 158–165). JMML is occasionally observed in NS carrying specific SHP2 pathogenic missense substitutions, (e.g., Y62D and T73I). In these patients, JMML often presents with a benign course which commonly regresses spontaneously even though a severe course has also been described (160). Transient benign myeloproliferative disorder (MPD) is also estimated to occur in up to 10% of all NS children. This disorder generally resolves spontaneously over months or years, although an estimated 10% of cases of NS/MPD may progress to JMML. Thus, MPD in NS should be closely monitored. Nearly all patients with NS and MPD carry mutations in *PTPN11* (162). The gain-of-function effect of these mutations is predicted to be intermediate, between that for NS without MPD (milder gain of function) and somatic mutations in JMML (stronger gain of function).

“Benign” proliferative conditions include multiple giant cell lesions (MGCL) and granular cell tumors (30). To date, few NS individuals with MGCL and mutations in *PTPN11* or *SOS1* have been identified (32), whereas MGCL is more frequently reported in patients with other RASopathies, such as in CFCS subjects carrying pathogenic variants of *BRAF* and *MEK1* (33).

In a cohort of 297 individuals carrying pathogenic *PTPN11* variants, cancer risk was estimated as 3.5-fold higher than in the general population. When considering all the RASopathies, (in a cohort of 632 individuals with molecularly defined NSSD), a 8.0-fold higher risk than controls has been found, although for CS a 42.4-fold increased risk was present (22, 31, 159). These data, again, stress the importance of genetic characterization in individuals affected with NS and other RASopathies.

Dedicated guidelines for cancer surveillance in patients with NS have yet to be developed (166). **Tables 3A**, **3B** and **3C** show the correlation between genotype and oncological risk in NS, which should be taken into consideration before and during rhGH treatment as different radiological follow-up is likely to be necessary for the various genotypes.

The more common NS-associated solid and soft tissue tumors include glioneuronal tumor and astrocytoma (33, 110, 112). While specific associations between a subset of *PTPN11* variants and pediatric hematological malignancies have been reported, the apparently higher incidence of *PTPN11* variants in NS individuals with tumors is likely to reflect the higher frequency of variants in this gene with respect to other genes. A significantly higher cancer risk is observed in CS, with typical association with bladder cancer and embryonal rhabdomyosarcoma.

The available data on rhGH and cancer in NS are reassuring (67), but underlying susceptibility to tumor growth should be considered when rhGH therapy is started (105, 127). Follow-up must be based on clinical symptoms, regular physical examinations and complete blood counts. Recent recommendations advise obtaining a brain MRI prior to initiating rhGH treatment in patients with NS, particularly in those with *PTPN11* mutations, as they appear to have a slightly increased risk for cranial neoplasms (105, 127, 166, 167).

The paucity of data on the long-term safety of rhGH therapy in patients with NS, especially regarding the risk of tumor development and tumor recurrence does not allow us to report a definitive consensus.

Data on IGF-I variations during rhGH treatment and variations in neoplastic risk in the medium and long term are largely lacking because rhGH treatment in non GHD patients was only introduced in 2007 after FDA approval.

Genotype characterization appears to be important in understanding the neoplastic risk for NS patients, and we stress the need to gather more data on different rhGH doses and differences between GHD Noonan and non-GHD Noonan.

### 4) Scoliosis and rhGH treatment

Besides short stature, skeletal findings in NS include kyphosis, lordosis, scoliosis, anterior chest wall anomalies and hand anomalies, such as syndactyly, brachydactyly, and cubitus valgus (168, 169). Chest wall anomalies are also extremely frequent, mostly represented by a superior pectus carinatum with an inferior pectus excavatum. Osteopenia/osteoporosis has not been frequently reported but was observed in a 2/26 individuals in one cohort (170).

Although an increased risk for development or progression of scoliosis is not apparent in NS patients treated with rhGH, this feature has not systematically been studied and continued surveillance is necessary (67). Romano et al. (62) reported 6 cases of scoliosis in 370 patients over 5.6 years of rhGH treatment, whereas Kirk et al. (73) reported 1 case of worsening kyphoscoliosis among 66 patients who were treated with rhGH for up to 6 years. In an observational study including a large number of NS subjects treated with rhGH (only 15% with a genetic diagnosis), three patients presented with scoliosis and three experienced arthralgia episodes. One patient needed spinal fusion surgery at 16.5 years of age, whereas the other 2 cases were considered non-serious and possibly unrelated to rhGH treatment; none of these patients had a diagnosis of scoliosis before rhGH treatment started (67). On the other hand, the condition of one patient affected with scoliosis prior to starting rhGH treatment, did not worsen during the treatment (67).

The data suggest that scoliosis-related outcomes are better than for TS during rhGH treatment (171). For example, out of 49 girls with Turner syndrome followed by Ricotti et al. (172), 29 exhibited scoliosis at baseline, and 9 additional individuals developed minor scoliosis during the 4-year follow-up, suggesting that these problems may be related to a worsening of pre-existing scoliosis (172).

### *5)* Arnold-Chiari I malformation and rhGH treatment

Arnold-Chiari malformation is commonly seen in RASopathies, and several cases have been reported in the medical literature in NS patients although the incidence is not known (28, 173). Arnold-Chiari is also observed in other medical conditions, including GHD (5–20%) (174), due to the underdevelopment of certain cranial bone structures.

## General concerns regarding GH treatment and further remarks

NS is a highly heterogeneous disorder, with variable clinical features and genetic complexity (1, 6–8, 13, 14), which must be taken into consideration when evaluating rhGH treatment in these patients. In addition to auxological and safety data, genetic data, often lacking, is essential for identifying patients at risk for specific side effects and complications during rhGH treatment. In many papers, diagnosis of NS is based only on clinical assessment (75), which makes it difficult to analyze the results and side effects for specific genotypes.

The presence of varying degrees of GHI in subjects with NS should not prevent clinicians from evaluating rhGH treatment, which should be individualized. Given the emerging data about neoplasms in NS patients, we recommend a conservative approach and judicious surveillance (8).

As in the general population, it is important to rule out GHD. The correct starting standard dose of rhGH has not been established in NS and high doses of rhGH are not recommended (175). In non-NS patients, some data suggest an increased risk for cardiovascular events (176) and increased incidence of secondary tumors (177) in children with a primary tumor who had been treated with rhGH during childhood and adolescence. Other authors have not found a significant increase in overall mortality in low-risk patients, such as those with isolated GHD (177). Evaluation of the GH-IGF-I axis could help determine the most appropriate starting dosage of rhGH. Even though most studies have not shown an increase in the incidence of neoplasms in NS patients treated with rhGH, there are no long-term studies specifically designed to address this issue. Since malignancies for patients with NS tend to involve multiple sites and develop throughout life, a routine tumor surveillance program should be implemented. In line with the authors of previously published studies (105, 110), we recommend that when rhGH therapy is initiated in NS patients, the possibility of performing a brain MRI is considered, particularly in subjects with *PTPN11* mutations who appear to have higher risk for CNS neoplasms.

Published data do not show any changes in myocardial function, or in ventricular wall thickness during rhGH treatment in NS patients but the lack of genetic data means that a definite conclusion cannot be reached. In children with a diagnosis of HCM a cautious approach and careful follow-up are necessary.

In conclusion, NS is a genetic disorder with substantial clinical variability, which in part is associated with the specific genes and mutations involved. Given the genetic and clinical complexity of the disorder and high prevalence of cardiac defects and malignancies, NS requires a multidisciplinary approach and follow-up.

The overall experience with rhGH treatment in most NS patients with short stature is reassuring; the data reveal few serious adverse effects. Therapy with rhGH increases HV in patients with NS, but firm conclusions regarding the effects of this therapy on near adult height and long-term health are not available. A better understanding of the causes of short stature, as well as response to rhGH treatment in NS, is needed and must be based on genetic characterization. Reliable large-scale and case-control studies are crucial in elucidating the long-term effects of rhGH treatment and defining the examinations necessary prior to treatment and in follow-up. Until we have more complete data, an individualized follow-up and close monitoring, also related to the cardiac, neoplastic and orthopedic risks, during rhGH treatment should be considered.

## Meyer University Hospital NoonanStudy group

Silvia Favilli, Giovanni Battista Calabri, Giulio Porcedda, Gaia Spaziani, Luciano De Simone, Elena Andreucci, Giovanna Traficante, Giorgia Mancano, Angelica Pagliazzi, Sara Bargiacchi, Giulia Gori.

## Authors contributions

All authors listed have made a substantial, direct, and intellectual contribution to the work, and approved it for publication.

## Funding

MT and SS have received speakers’ bureau honoraria from Novo Nordisk. The other authors do not have any financial or non-financial competing interests in relation to this manuscript.

## Conflict of interest

The authors declare that the research was conducted in the absence of any commercial or financial relationships that could be construed as a potential conflict of interest.

## Publisher’s note

All claims expressed in this article are solely those of the authors and do not necessarily represent those of their affiliated organizations, or those of the publisher, the editors and the reviewers. Any product that may be evaluated in this article, or claim that may be made by its manufacturer, is not guaranteed or endorsed by the publisher.
